# GC-MS and GC-IMS Comprehensive Analysis of Volatile Compounds in the Peel and Pulp of Six Lemon Varieties and Their Interactions with Olfactory Receptors: Molecular Docking and Molecular Dynamics Simulations Studies

**DOI:** 10.3390/foods15101710

**Published:** 2026-05-13

**Authors:** Chengcheng Liu, Yang Wu, Lingzhen Lv, Xiang Sun, Qiuju Dai, Yijun Wang, Shilin Chen, Zhixiang Liu

**Affiliations:** 1 School of Pharmacy, Chengdu University of Traditional Chinese Medicine, Chengdu 611137, China; l18674128836@163.com (C.L.);; 2 Institute of Herbgenomics, Chengdu University of Traditional Chinese Medicine, Chengdu 611137, China

**Keywords:** *Citrus × limon*, GC-MS, GC-IMS, olfactory receptors, molecular docking, molecular dynamic simulations

## Abstract

The aroma of lemon is an important indicator of its quality, but there is still a lack of comparative research on the flavor components of different varieties of lemon and the interaction between olfactory receptors. This study conducted a combined analysis of volatile components in the peel and flesh of six varieties of lemons using GC-MS and GC-IMS. The random forest algorithm in machine learning was used to screen differential metabolites and perform molecular docking with all 389 olfactory receptors to analyze their potential interaction relationships. Hydrophobic interactions and hydrogen bonds are the most common interactions between odor molecules and olfactory receptors, and amino acids such as PHE, TYR, ASN, and HIS have the highest frequency of interaction. These findings offer crucial insights into the molecular mechanisms underlying lemon flavor. They also furnish a theoretical foundation for enhancing and utilizing lemon flavor quality.

## 1. Introduction

Lemon (*Citrus × limon*), a fruit of the Rutaceae family, is globally esteemed for its distinct flavor, nutritional value, and wide-ranging applications in the food, cosmetic, and pharmaceutical industries [1]. The characteristic aroma of lemon is a critical indicator of its quality and consumer acceptance, arising from a complex mixture of volatile organic compounds (VOCs) including terpenes, esters, aldehydes, and alcohols [2]. It is well-established that different lemon varieties exhibit unique sensory profiles, a phenomenon largely attributed to quantitative and qualitative differences in their VOC compositions [3]. Previous research has identified compounds such as (+)-limonene, citral, and myrcene as key contributors to the typical citrus aroma [4]. However, a comprehensive comparative analysis of the volatile profiles in both the peel and pulp across multiple lemon cultivars is still limited. More importantly, the molecular mechanisms that govern how these characteristic aroma compounds are perceived by the human olfactory system remain largely unelucidated.

To comprehensively characterize the aroma profile of complex matrices like lemon, advanced analytical techniques are required. Gas chromatography-mass spectrometry (GC-MS) is a cornerstone for the identification and quantification of volatile compounds. However, to achieve higher sensitivity and resolve co-eluting compounds, gas chromatography-ion mobility spectrometry (GC-IMS) has emerged as a powerful complementary tool. GC-IMS combines the high-resolution separation of gas chromatography with the rapid, sensitive detection of ion mobility spectrometry, allowing for the creation of unique two-dimensional fingerprints of a sample’s volatile profile [5]. Compared to conventional GC-MS, GC-IMS offers superior separation efficiency, faster response times, and minimal sample pretreatment requirements [6]. This technique has gained widespread application in food chemistry, environmental monitoring, clinical diagnostics, and herbal medicine analysis over the past decade [7,8,9,10].

Olfactory receptors (ORs), phylogenetically classified within the class A rhodopsin-like subgroup of G protein-coupled receptors (GPCRs), constitute the largest family of chemosensory proteins in vertebrates [11]. These canonical seven-transmembrane domain receptors mediate odorant detection through their specific expression in olfactory sensory neurons of the olfactory epithelium, initiating the olfactory signal transduction cascade. The olfactory system can detect and distinguish thousands of odor compounds, a feat achieved through complex recognition mechanisms of olfactory receptors. Initially, ref. [12] discovered the OR gene family, which provided the groundwork for comprehending the molecular mechanisms of olfactory perception. Subsequently, ref. [13] further proposed the concept of Combined OR Code, which explains how olfactory perception can distinguish a vast array of odors through a limited number of ORs. The core of this concept includes the following two points: (1) An odor molecule can activate multiple ORs. For example, vanillin can simultaneously activate OR7D4, OR5AC2, and OR10G7, rather than acting on a specific OR alone [14,15]. (2) An OR can respond to multiple different odor molecules. For instance, OR1A1 may respond to both citronellal and citronellol [16]. While this combinatorial system provides immense discriminatory power, the specific ligand-receptor pairings for most human ORs remain unknown, hindering a mechanistic understanding of flavor perception.

Given the challenges of experimentally deorphanizing the entire OR repertoire, computational approaches such as molecular docking and molecular dynamics (MD) simulations have become indispensable tools in flavor science [17]. Molecular docking provides a static prediction of the binding affinity and preferred conformation of a ligand within a receptor’s binding pocket. While insightful, these static models do not capture the dynamic nature of protein-ligand interactions. MD simulations complement this by simulating the atomic movements of the complex over time, allowing for the validation of docking poses and the assessment of binding stability, conformational changes, and key intermolecular forces like hydrogen bonds and hydrophobic interactions [18]. The integration of these methods has been successfully used to explore the interactions between characteristic aroma compounds and ORs in tea, coffee, and various fruits [19,20,21].

Therefore, this study was designed to integrate advanced analytical and computational methods to unravel the flavor chemistry of lemon. The primary objectives were: (i) to comprehensively analyze and compare the volatile compound profiles in the peel and pulp of six distinct lemon varieties using a combination of GC-MS and GC-IMS; (ii) to identify key differential aroma compounds using the random forest machine learning algorithm; and (iii) to investigate the molecular recognition mechanisms by performing molecular docking of these characteristic compounds against the complete human OR repertoire, followed by MD simulations of the most significant ligand-receptor pairs. This work aims to elucidate the molecular basis of lemon aroma perception, providing a theoretical framework for quality assessment, varietal improvement, and the targeted enhancement of lemon-derived products.

## 2. Materials and Methods

### 2.1. Materials, Reagents and Instruments

Six varieties of lemon, including Eureka, Bearss, Lisbon, Fino, Femminello, and Verna, were harvested at commercial maturity from the official germplasm resource nursery in Ziyang, Sichuan Province, China (30°10′ N, 105°44′ E) (Table 1). Fruits were selected for uniformity in size and color, and absence of any physical damage or disease. A C7–C40 n-alkane mixture, used for calculating Retention Indices (RIs), and all analytical standards for volatile compounds were purchased from Sigma-Aldrich (Shanghai, China). Saturated sodium chloride solution was prepared using analytical grade NaCl. High-purity helium (≥99.999%) and nitrogen (≥99.999%) were sourced from a local supplier. The analysis was performed using an Agilent 8890-7000E GC-MS/MS system (Agilent Technologies, Santa Clara, CA, USA) equipped with a CTC Analytics PAL3 autosampler (CTC Analytics AG, Zwingen, Switzerland) for headspace solid-phase microextraction (HS-SPME). A Retsch MM400 ball mill (Haan, Germany) was used for cryogenic grinding. A 120 µm DVB/CWR/PDMS SPME Arrow fiber (CTC Analytics AG, Zwingen, Switzerland) was employed for extraction. GC-IMS analysis was conducted on a FlavourSpec^®^ Gas-Phase Ion Mobility Spectrometer (G.A.S., Dortmund, Germany).

### 2.2. Headspace Solid Phase Micro-Extraction Gas Chromatography-Mass Spectrometry (HS-SPME-GC-MS) Analysis

Lemon samples were separated into peel and pulp, immediately frozen in liquid nitrogen, and cryogenically ground into a fine powder using a ball mill (MM400, Retsch GmbH, Haan, Germany). Approximately 500 mg of the frozen powder was accurately weighed into a 20 mL headspace vial. Saturated NaCl solution was added to the vial, followed by 20 µL of internal standard solution (10 µg/mL, a mixture of six 2-ketones: 2-butanone, 2-pentanone, 2-hexanone, 2-heptanone, 2-octanone, and 2-nonanone). The vials were sealed and subjected to automated headspace solid-phase microextraction (HS-SPME). Extraction was performed using a 120 µm DVB/CWR/PDMS SPME Arrow fiber (Agilent Technologies, Santa Clara, CA, USA). The samples were equilibrated at 60 °C for 5 min with shaking, followed by headspace extraction for 30 min at 60 °C. Prior to sampling, the fiber was conditioned at 250 °C for 5 min in a Fiber Conditioning Station. After extraction, the volatile organic compounds (VOCs) were desorbed from the fiber coating in the GC injection port at 250 °C for 5 min in splitless mode. Chromatographic separation was performed on an Agilent 8890 GC system coupled to a 7000E mass spectrometer. A DB-5MS capillary column (30 m × 0.25 mm × 0.25 µm, Agilent J&W Scientific, Folsom, CA, USA) was used. High-purity helium (≥99.999%) served as the carrier gas at a constant flow rate of 1.2 mL/min. The oven temperature program was initiated at 40 °C (held for 3.5 min), increased to 100 °C at 10 °C/min, then to 180 °C at 7 °C/min, and finally to 280 °C at 25 °C/min, where it was held for 5 min. Mass spectra were recorded in electron impact (EI) ionization mode at 70 eV. The ion source, quadrupole, and transfer line temperatures were set to 230 °C, 150 °C, and 280 °C, respectively. Crucially, the mass spectrometer operated in Selected Ion Monitoring (SIM) mode. Qualitative identification was performed using the MWDB database based on retention times and specific quantitative/qualitative ion transitions. Quantification was achieved using the peak area of the selected quantitative ion for each compound, corrected by the internal standard.

### 2.3. Headspace Gas Chromatography—Ion Mobility Spectrometry (HS-GC-IMS) Analysis

Analyses were performed using a FlavourSpec^®^ Gas-Phase Ion Mobility Spectrometer (G.A.S., Dortmund, Germany) equipped with a CTC Analytics PAL3 autosampler (Zwingen, Switzerland). Sample preparation involved accurately weighing 0.1 g of lemon sample into a 20 mL headspace vial. The samples were incubated at 60 °C for 30 min with an oscillator speed of 500 rpm to ensure the release of volatile compounds. Following incubation, 100 µL of the headspace gas was injected automatically in non-split mode with the injection needle temperature maintained at 85 °C to prevent condensation. Chromatographic separation was achieved using a DB-WAX capillary column (15 m × 0.53 mm, 1.0 μm, Restek Corporation, Bellefonte, PA, USA) maintained at 60 °C. High-purity helium (purity ≥ 99.999%) was employed as the carrier gas. The gas flow rate was programmed as follows: initially maintained at 2.00 mL/min for 2 min, linearly increased to 10.00 mL/min over 8 min, further increased to 100.00 mL/min over the next 10 min, and finally held at 100.00 mL/min for 10 min. The total run time was 30 min. For the IMS detection, a tritium source (^3^H) was used for ionization. The drift tube was 53 mm in length and operated at a constant temperature of 45 °C with an electric field intensity of 500 V/cm. High-purity nitrogen (purity ≥ 99.999%) was used as the drift gas at a flow rate of 150 mL/min. The system operated in positive ion mode.

### 2.4. Molecular Docking and MD Simulations

The three-dimensional (3D) structures of the identified differential volatile compounds (ligands) were retrieved from the PubChem database in SDF format. To ensure structural reliability, geometry optimization and format conversion to PDBQT were performed using OpenBabel 3.1.1, during which polar hydrogen atoms were added and rotatable bonds were defined. The amino acid sequences of 389 functional human olfactory receptors (ORs) were obtained from the UniProt database. Their 3D protein structures were predicted and downloaded from the AlphaFold Protein Structure Database. The potential ligand-binding pockets of these receptors were predicted using the CavityPlus web server (Table S1). Prior to docking, the receptor structures were pre-processed using AutoDockTools-1.5.6. This process involved the removal of water molecules, the addition of polar hydrogen atoms, the calculation of Gasteiger charges, and the assignment of AD4 atom types. The prepared receptor structures were subsequently saved in PDBQT format.

Molecular docking was performed using AutoDock Vina (v1.1.2) to predict the binding modes and affinities between the volatile compounds and ORs. A semi-flexible docking protocol was employed, wherein the receptor was kept rigid while the ligands were allowed full torsional flexibility. The exhaustiveness parameter was set to 8 to balance computational efficiency and search depth. The resulting ligand-receptor complexes were ranked based on their binding affinity scores (kcal/mol). To elucidate the molecular recognition mechanisms, the interactions (including hydrophobic interactions, hydrogen bonds, π-stacking, etc.) within the best-scored complexes were analyzed and visualized using the Protein-Ligand Interaction Profiler (PLIP) web server and PyMOL 3.1.3.

To validate the stability of the docking predictions and explore the dynamic behavior of the ligand-receptor complexes, 100 ns molecular dynamics (MD) simulations were conducted using the GROMACS 2019.6 package. The top-scoring odorant-receptor complexes identified from the docking studies were used as initial structures. System Construction: The receptor was embedded into a pre-equilibrated 1-palmitoyl-2-oleoyl-sn-glycero-3-phosphatidylcholine (POPC) lipid bilayer, with the protein orientation determined using the OPM (Orientations of Proteins in Membranes) database. The system was solvated in a periodic hexagonal box using the TIP3P water model and neutralized by adding 0.15 M NaCl ions to mimic physiological conditions. The CHARMM36m force field was applied to model the protein, lipids, and ions, while the ligand topology and parameters were generated using the CGenFF server (CHARMM General Force Field). Simulation Protocol: The system underwent energy minimization for 10,000 steps (combining 5000 steps of steepest descent and 5000 steps of conjugate gradient algorithms) to remove steric clashes. This was followed by a two-step equilibration phase: a 1000 ps NVT ensemble simulation (310 K) to stabilize the temperature, and a 1000 ps NPT ensemble simulation (1 bar) to stabilize the pressure, with harmonic position restraints applied to the protein and ligand heavy atoms. Finally, a 100 ns production MD run was performed for each complex without restraints, using a time step of 2 fs. Parameters and Analysis: The Particle Mesh Ewald (PME) method was utilized to compute long-range electrostatic interactions with a cut-off of 12 Å. The LINCS algorithm was employed to constrain bond lengths involving hydrogen atoms. Trajectory analysis was performed to calculate the Root Mean Square Deviation (RMSD), Root Mean Square Fluctuation (RMSF), and hydrogen bond occupancy over the simulation time using GROMACS 2024.4 built-in tools and Origin software 2024.

### 2.5. Data Processing and Statistical Analysis

The raw data acquired from HS-SPME-GC-MS were processed using Agilent MassHunter software B.09.00 for peak deconvolution, alignment, and integration. To ensure dataset integrity, missing values were imputed using the K-nearest neighbors (KNN) algorithm. Subsequently, feature filtration was performed based on the quality control (QC) samples; metabolites with a Coefficient of Variation (CV) exceeding 0.5 were removed to maintain high data reliability. The relative content of volatile compounds was calculated using the internal standard semi-quantitative method. For GC-IMS data, the VOCal software (version 0.4.03, G.A.S., Dortmund, Germany) was utilized for qualitative and quantitative analysis. The retention indices (RIs) were calibrated using a mixture of n-ketones (C4–C9) as external references, and compound identification was achieved by matching RIs and ion migration times against the NIST 2020 and IMS databases. Referring to the method described by [22], the ROAV of each component was calculated, based on which the main volatile components were determined. It is generally believed that components with ROAV ≥ 1 are the key flavor compounds of the analyzed sample, while components with 0.1 ≤ ROAV < 1 have an important modifying effect on the overall flavor of the sample.

All experiments were performed in triplicate, and data were expressed as mean ± standard deviation (SD). One-way analysis of variance (ANOVA) followed by Duncan’s multiple range test was conducted to evaluate significant differences (*p* < 0.05) in volatile compound concentrations among different lemon varieties using SPSS 23.0 software (IBM Corp., Armonk, NY, USA). Multivariate statistical analyses were performed using the Metware Cloud platform (https://cloud.metware.cn (accessed on 7 April 2025)). Principal Component Analysis (PCA) was employed as an unsupervised method to visualize the overall distribution and clustering trends of the samples. Partial Least Squares Discriminant Analysis (PLS-DA) was applied to maximize the separation between groups. To identify key biomarkers distinguishing the lemon varieties, the Random Forest algorithm was utilized to screen for differential metabolites based on the Mean Decrease Accuracy (MDA) scores. General data visualization, including the plotting of MD simulation trajectories (RMSD, RMSF, etc.), was performed using Origin 2021 (OriginLab, Northampton, MA, USA) and Microsoft Excel 2021.

## 3. Results and Discussion

### 3.1. HS-SPME-GC-MS Analysis

**Figure 1:**
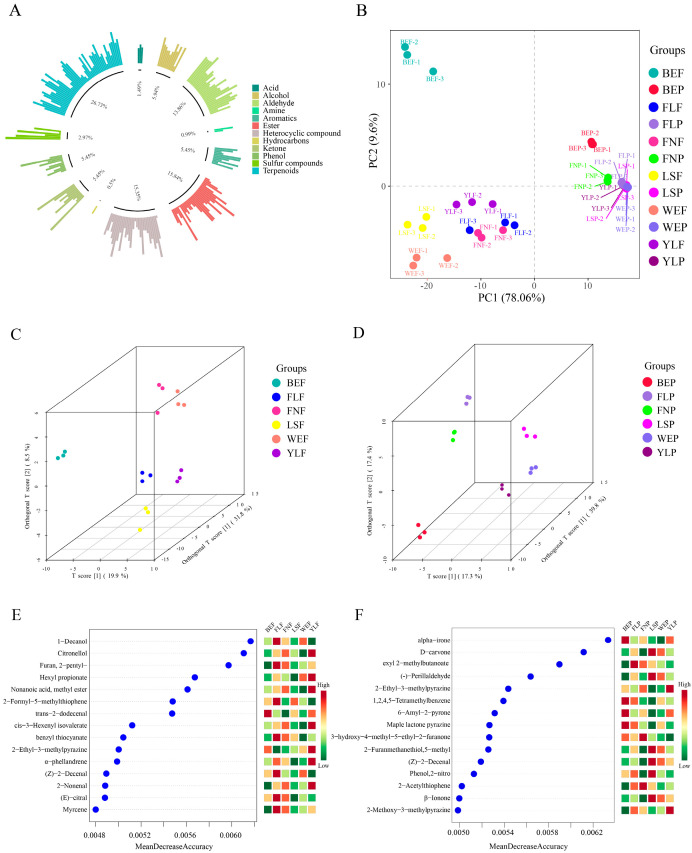
Types of volatile compounds detected in lemon by GC-MS analysis (**A**); PCA of volatile compounds in different varieties of lemon (**B**). PCoA analysis of volatile compounds in lemon pulp (**C**) and peel (**D**); The differential metabolites detected by GC-MS in the fruit pulp (**E**) and fruit peel (**F**) were obtained through random forest screening.

Through GC-MS analysis, a total of 202 volatile compounds with rOAV values greater than 1 were identified, suggesting that these compounds play a crucial role in determining the sample’s flavor characteristics. In these volatile compounds, there are 54 terpenoids, 32 esters, 31 heterocyclic compounds, 28 aldehydes, 12 alcohols, 11 phenols, 11 ketones, 11 aromatics, 6 sulfur compounds, 3 acids, 2 amines, and 1 hydrocarbon (Figure 1A, Table S2). 1-p-Menthene-8-thiol is the compound with the highest rOAV value, exceeding 10^8^. Studies have shown that it can activate OR2W1 and OR8H1 receptors [23,24]. The relative content of terpenoids, alcohols, ketones, and phenols in the pulp was higher than that in the peel, while the relative content of esters, heterocyclic compounds, and aldehydes was lower than that in the peel (Table S3), indicating that lemon fruit pulp and fruit peel have different flavors. Studies have shown that compounds such as terpenes, esters, and heterocycles play a crucial role in the flavor of fruits and vegetables [25]. Principal component analysis (PCA) was performed on the volatile components of all samples. The results showed that the first two principal components explained 87.66% of the total variance in the dataset, with PC1 accounting for 78.06% and PC2 for 9.60%. The separation of the peel and pulp along the PC1 axis indicated significant differences in the volatile components between the lemon peel and pulp (Figure 1B). This is expected, as the peel (specifically the flavedo) is the primary location of oil glands where many characteristic volatiles are synthesized and stored, while the pulp’s profile is more influenced by water-soluble compounds and products of enzymatic reactions. Therefore, in subsequent analyses, the data for the lemon peel and pulp were analyzed separately.

Partial least squares discriminant analysis (PLS-DA), a supervised discriminant analysis method, is widely employed to classify subjects based on the observed or measured values of multiple variables [26]. PLS-DA results showed that the volatile components of peel and pulp of different varieties of lemon were significantly different (Figure 1C,D). The BEP and BEF groups are relatively far from other groups, indicating that the volatile compounds in bearss lemon fruit are more unique than those in other varieties of lemons. To further identify the specific differential metabolites in lemon fruits of different varieties, the algorithm of random forest was employed to screen the differential metabolites between groups [27]. Utilizing the random forest algorithm in machine learning, 15 differential metabolites were respectively screened out from the volatile metabolites of the pulp (Figure 1E) and peels (Figure 1F) of six lemon varieties. Among the 15 differential metabolites in the lemon pulp, there were four terpenoids, three heterocyclic compounds, three esters, three aldehydes, one alcohol, and one sulfur compound. In the BEF group, the relative content of trans-2-dodecenal is higher than that of other groups; There are many characteristic volatile components in the FLF group, including 1-Decanol, Furan, 2-pentyl-, 2-formyl-5-methylthiophene, benzyl thiocyanate, (Z)-2-decanal, (E)-citral, and Myrcene; There are also many characteristic components in the YLF group, including Citronellol, Hexyl propionate, Nonanoic acid, methyl ester, cis-3-Hexenyl isovalerate, 2-Ethyl-3-methylpyrazine, α-phellandrene and 2-Nonenal. There are fewer characteristic components in the flesh of other varieties of lemons. The results showed that nearly half of the 15 different metabolites in the fruit peel belonged to the category of heterocyclic compounds, although the proportion of heterocyclic compounds to the total number of compounds was only 15.35%. Studies have shown that heterocyclic compounds, which contribute significantly to meaty flavors through their extremely low odor thresholds and key roles in meat flavor formation [28]. Additionally, these included three terpenoids, two aldehydes, one aromatic compound, one ester, and one phenol. The BEP group has more characteristic volatile compounds, including alpha-irone, 1,2,4,5-tetramethylbenzene, 6-amyl-2-pyrone and maple lactone pyrazine; there are also many characteristic volatile compounds in the LSP group, including D-carvone, (−)-perillaldehyde, 5-methyl-2-furanmethanethiol, (Z)-2-decenal, 2-nitrophenol, and β-ionone; ethyl 2-methylbutanoate can be used as a characteristic volatile component of FLP; 3-hydroxy-4-methyl-5-ethyl-2-furanone and 2-acetylthiophene can serve as characteristic volatile compounds of FNP; 2-methoxy-3-methylpyrazine is a characteristic volatile compound of the YLP group. Only two compounds were common between the peels and pulp differential metabolites, namely 2-Ethyl-3-Methylpyrazine (a heterocyclic compound) and (Z)-2-Decenal (an aldehyde). GC-MS analysis of volatile compounds in different lemon varieties identified 28 differential metabolites, 12 of which have been previously reported to interact with olfactory receptors (Table S4). This existing knowledge provides a strong foundation for our hypothesis that these differential compounds are not just chemical markers but are functionally relevant to lemon flavor perception, justifying their further investigation through computational methods.

In conclusion, the GC-MS analysis successfully identified 202 volatile compounds contributing to lemon aroma, with clear compositional differences observed between the peel and pulp. The random forest algorithm screened 28 differential metabolites across six varieties, among which 12 have been previously reported to interact with olfactory receptors. These findings establish a robust chemical foundation for understanding variety-specific lemon flavors and support the subsequent investigation of odorant-receptor interaction mechanisms through molecular docking.

### 3.2. HS-GC-IMS Analysis

To obtain a more holistic view of the lemon volatile profile, we employed HS-GC-IMS, a technique renowned for its high sensitivity and ability to create unique two-dimensional fingerprints based on both gas chromatographic retention time and ion mobility drift time. This approach is particularly powerful for resolving co-eluting compounds and detecting trace volatiles that might be missed by GC-MS alone. The analysis identified 82 signal peaks, corresponding to 74 distinct compounds (Table 2). Crucially, only 17 of these compounds overlapped with the GC-MS results, underscoring the complementary nature of the two techniques and the value of a multi-modal analytical strategy for comprehensive flavoromics.

**Figure 2:**
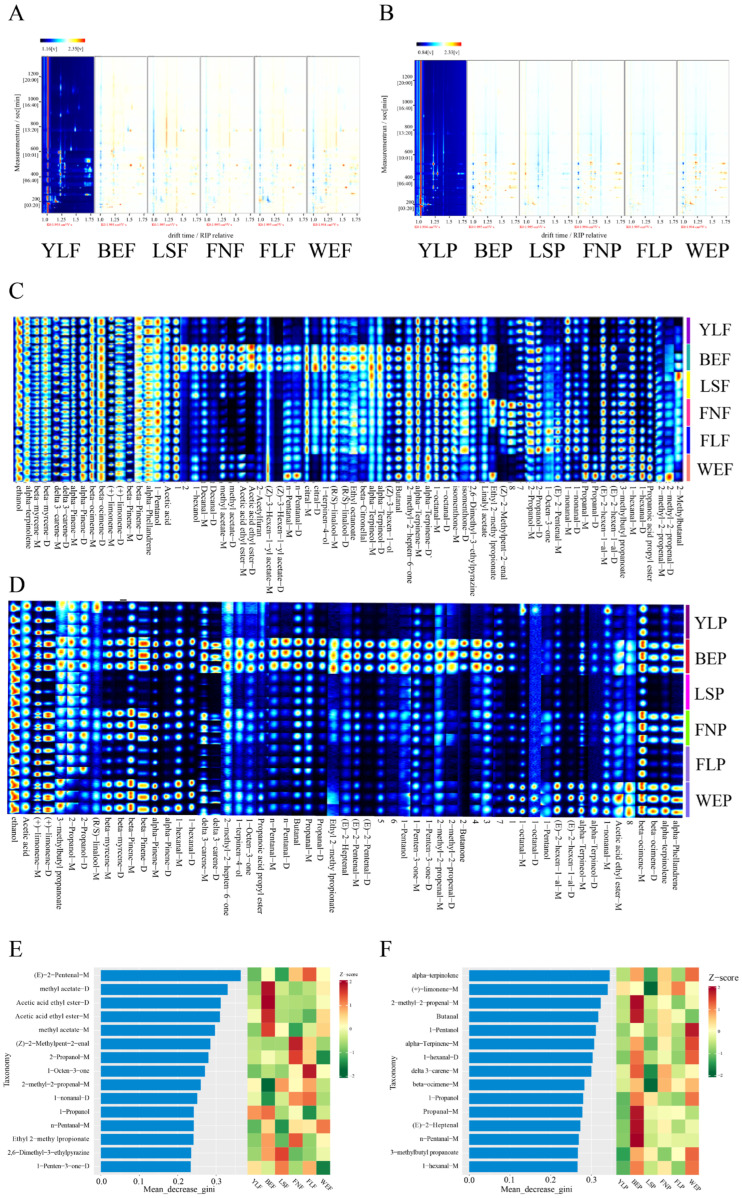
2D topographic images with differential comparison mode of 6 lemon samples (pulp, (**A**); peel, (**B**)); Fingerprint spectra of volatile compounds in the pulp (**C**) and peel (**D**) of different varieties of lemon using Gallery Plot characterization; The differential metabolites detected by GC-IMS in the fruit pulp (**E**) and fruit peel (**F**) were obtained through random forest screening.

The three-dimensional spectrum of GC-IMS elucidates the variations in volatile organic compounds among different lemon fruit and peel varieties (Figure 2A). The three coordinate axes denote migration time (*x*-axis), retention time (*y*-axis), and signal peak intensity (*z*-axis). To facilitate observation, a top view is provided for comparison (Figure 2B). The spectral background is blue, with each signal spot to the right of the reactant ion peak representing a volatile compound. A single compound may generate multiple signals or spots (monomers or dimers), contingent upon its concentration and properties. Some compounds may produce multiple signals due to varying concentrations. When passing through the drift zone, multiple signals of a single compound may be observed due to the formation of adducts between the analyzed ion and neutral molecules. The color of the signal spots ranges from white (low concentration) to red (high concentration). The results showed that most of the volatile compounds in the pulp and peel of different varieties of lemon were located in the region with residence time of 100~800 s and drift time of 1.0~1.75 s. The spectrum contains many signal peaks, indicating that lemon samples contain rich volatile compounds.

Further comparisons of the volatile substances in lemon pulp were conducted, and fingerprint analysis was performed on all volatile substances (Figure 2C,D). The results showed that the contents of ethanol, terpinolene, myrcene, 3-carene, and α-pinene varied little among different lemon varieties. The contents of limonene, β-pinene, phellandrene, 1-pentanol, and acetic acid were higher in YLF. BEF contained more characteristic VOCs, including higher contents of hexanol, decanal, methyl acetate, acetic acid ethyl ester, 2-acetylfuran, n-pentanal, (R/S)-linalool, and ethyl octanoate than other varieties. The contents of alpha-terpinene, 1-octanal, and isomenthone were higher in LSF. The contents of ethyl 2-methylpropionate, (Z)-2-methylpent-2-enal, and 2-propanol were higher in FNF. The contents of 1-octen-3-one, (E)-2-pentenal, and 1-nonanal were higher in FLF. The contents of propanal, (E)-2-hexen-1-al, 1-hexanal, and propanoic acid propyl ester were higher in WEF. The results of the GC-IMS analysis of the lemon peel from different varieties showed that the contents of (R/S)-linalool, (+)-limonene, beta-myrcene, β-pinene, α-pinene, 1-hexanal, and delta 3-carene were higher in BEP, FNP, and WEP, and lower in the pulp of the other three varieties. In addition, in BEP, the contents of propanoic acid propyl ester, n-pentanal, butanal, propanal, ethyl 2-methylpropionate, and (E)-2-heptenal were much higher than in other groups. Similarly, in WEP, the contents of 1-octanal, n-pentanal, and alpha-terpinene were much higher than in other groups. Overall, the VOCs were lower in YLP, LSP, and FLP of the lemon pulp.

Of the 24 differential metabolites identified by GC-IMS (Figure 2E,F), only seven have been previously reported to interact with olfactory receptors (Table 3), and the interactions of the remaining compounds with olfactory receptors remain largely unknown. Similar to the interaction between differential metabolites screened by GC-MS analysis and olfactory receptors, most of the differential metabolites screened by GC-IMS analysis have an interaction relationship with wide tuning olfactory receptors.

In conclusion, the GC-IMS analysis complemented the GC-MS results by identifying 74 distinct volatile compounds, with only 17 overlapping between the two techniques, highlighting the value of a multi-modal analytical strategy. Fingerprint analysis revealed variety-specific volatile profiles, and 24 differential metabolites were screened, among which seven have documented interactions with olfactory receptors. These results enhance our understanding of the comprehensive volatile composition of lemon and identify additional candidate compounds for molecular docking investigations.

### 3.3. Molecular Docking Analysis

**Figure 3:**
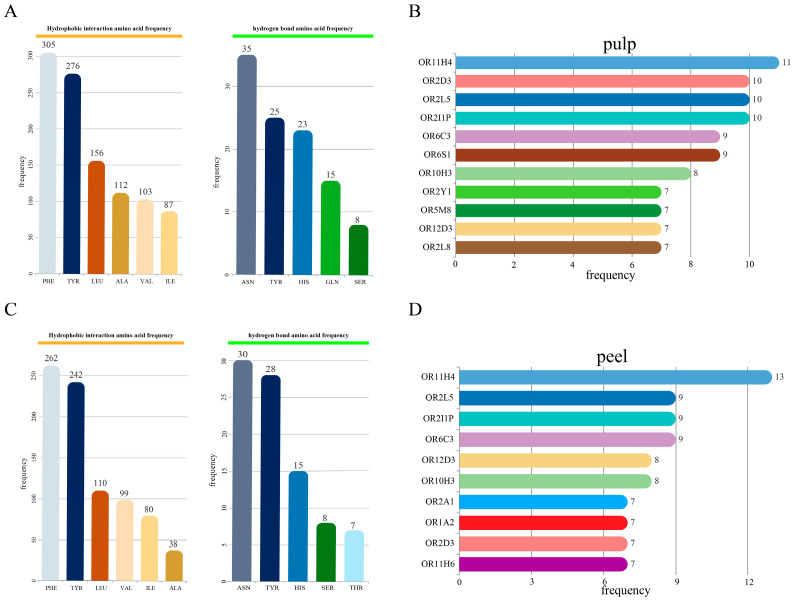
The frequency of amino acids in receptor ligand pairs in fruit pulp (**A**) and fruit peel (**C**). The differential metabolites between fruit pulp (**B**) and fruit peel (**D**) were docked with 389 olfactory receptor molecules, and the frequency of binding to the top 2–3 olfactory receptors was determined.

To bridge the gap between the identified volatile compounds and their biological perception, we performed a large-scale molecular docking analysis. The differential metabolites from both GC-MS and GC-IMS were docked against the entire repertoire of 389 functional human olfactory receptors. This comprehensive computational screening allows for the prediction of potential odorant-receptor pairs and the elucidation of the underlying interaction forces. The ligand-receptor pairs with the top 2–3 binding energies were selected for further analysis. The analysis included 109 ligand receptor pairs identified by GC-MS screening of differential metabolites and olfactory receptors in different varieties of lemon pulp, and 99 ligand receptor pairs identified by GC-IMS screening of differential metabolites and olfactory receptors. The analysis results show that the docking binding energies ranged from −3.5 to −7.8 kcal/mol. The binding forces between the small molecules and olfactory receptors included four types: hydrophobic interactions, hydrogen bonds, π-stacking, and salt bridges. Hydrophobic interactions and hydrogen bonds were the main interacting forces. Among them, the hydrophobic amino acid phenylalanine (PHE) was the most frequently occurring amino acid in hydrophobic interactions, asparagine (ASN) was the most frequently occurring amino acid in hydrogen bonds, and tyrosine (TYR) ranked second in both types of interactions (Figure 3A, Table S5). In the molecular docking results of differential metabolites in the pulp with olfactory receptors, the most frequently occurring receptors include OR11H4, OR2I1P, OR2L5, OR2D3, OR6S1, OR6C3, OR10H3, OR2L8, OR12D3, OR5M8, and OR2Y1 (Figure 3B).

Similarly, in the molecular docking results of differential metabolites and olfactory receptors obtained through GC-MS and GC-IMS analysis in lemon peels of different varieties, a total of 196 ligand receptor pairs were included in the analysis. The binding energy is between −3.5 and −8.4 kcal/mol. The results showed that the interaction forces between odor molecules and olfactory receptors include five types: hydrophobic interactions, hydrogen bonds, π-stacking, salt bridges, and π-cation interactions. Among them, hydrophobic interactions and hydrogen bonds are the most significant interaction forces. Similar to the differential metabolites and olfactory receptor interactions in lemon peels of different varieties, PHE and TYR are the most frequently occurring amino acids in hydrophobic interactions, while ASN and TYR are the most frequently occurring amino acids in hydrogen bonds (Figure 3C, Table S6). In the molecular docking results of differential metabolites in the peel with olfactory receptors, the most frequently occurring receptors include OR11H4, OR6C3, OR2I1P, OR2L5, OR10H3, OR12D3, OR11H6, OR2D3, OR1A2, and OR2A1 (Figure 3D). Overall, the results of molecular docking suggest that the characteristic volatile components in different varieties of lemons may have close interactions with olfactory receptors such as OR11H4, OR2I1P, OR2L5, OR6C3, and OR2D3.

**Figure 4:**
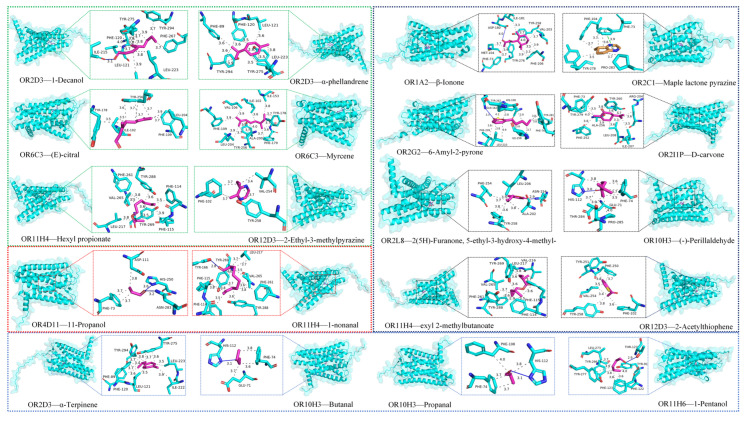
Molecular docking of 20 compounds of different aroma types with olfactory receptors. The gray dashed line represents the hydrophobic interaction between olfactory receptors and odor molecules; The blue implementation represents hydrogen bonding; The yellow dashed line represents Salt Bridges; The green dashed line represents π-Stacking.

All the differential metabolites were annotated into 20 aroma types, including green, ethereal, and fatty, etc. For the compounds of each aroma type, one compound was randomly selected (Table S7). The molecular docking of these 20 compounds with the olfactory receptors that have the minimum binding energy was analyzed in detail (Figure 4). The results show that hydrophobic interactions and hydrogen bonds are the main interaction forces. The distance between two hydrophobic molecules or groups is within the reasonable range of 2.94 Å–3.99 Å (Table S8). However, the distance between hydrogen-bond donors and acceptors is mostly over 3.0 Å, indicating a weak hydrogen-bonding interaction between odor molecules and olfactory receptors.

Studies have shown that a specific rose extract and its main components, phenylethyl alcohol and phenyl propyl alcohol, can activate olfactory receptors like OR11H4, effectively reducing skin stress biomarkers caused by stress-induced skin fatigue [43]. Another study suggests that OR11H4 may be involved in the giant panda’s specific recognition of Isovaleric acid [44]. In our study, among the 389 olfactory receptors, OR11H4 had the lowest binding energy with three differential metabolites in the molecular docking results, all with binding energies below −6 kcal/mol, indicating a strong binding effect and suggesting its potential role in the olfactory perception of lemon volatile substances. OR1A2, classified as a narrow-range tuning receptor, has been found to play a key role in recognizing the characteristic aroma in orange juice [45]. β-Inone has been reported to interact with OR1A2 [16] and exhibits the lowest binding energy among these 20 receptor ligand pairs, indicating a strong affinity between them. Analysis shows that there are 13 hydrophobic interactions and one hydrogen bonding force between them. However, the bond length of the hydrogen bond exceeds 3.5 Å, and it is generally believed that the bond length of the hydrogen bond is relatively stable in the range of 1.8–3.0 Å. When it exceeds 3.5 Å, the hydrogen bond interaction will significantly weaken, so it can be inferred that there is a very weak hydrogen bond interaction between them [46]. However, this preliminary molecular docking result still needs to be further validated through MD simulations and other analyses to gain a more comprehensive understanding of the interaction mechanism between β-Inone and OR1A2.

In conclusion, the molecular docking analysis revealed that hydrophobic interactions and hydrogen bonds are the predominant forces governing the binding of lemon volatile compounds to olfactory receptors. Amino acid residues PHE, TYR, and ASN exhibited the highest interaction frequencies, while receptors OR11H4, OR2I1P, OR2L5, OR6C3, and OR2D3 showed the strongest binding affinities across multiple differential metabolites. The lowest binding energy was observed between β-Ionone and OR1A2, suggesting a potentially significant role in lemon aroma perception. These docking predictions provide essential insights into the molecular recognition mechanisms and establish a basis for subsequent dynamic validation through MD simulations.

### 3.4. MD Simulations Analysis

While molecular docking provides a static snapshot of the most probable binding pose, the interaction between an odorant and an olfactory receptor is a dynamic process involving conformational adjustments of both ligand and protein [47]. To gain deeper insights into this mechanism, MD simulations were performed to investigate intermolecular interactions, based on the thermodynamic and kinetic properties of the molecular system [48]. To evaluate the overall stability of the binding system, the RMSD was calculated for the entire receptor-ligand complex. RMSD quantifies structural differences between molecular conformations by measuring the root-mean-square of atomic position deviations [49]. It evaluates system stability and conformational changes in MD simulations. Low RMSD values indicate structural consistency, while significant fluctuations suggest dynamic rearrangements, commonly applied to ligand-receptor complexes [50]. In our MD results, the RMSD curves of the receptor-ligand complexes tended to be stable, indicating that the olfactory receptors and ligands maintained a stable bound state throughout the simulation (Figure S1) [51]. RMSF analysis was conducted to assess the local flexibility of the receptor-ligand complex residues. High RMSF values correspond to flexible regions (such as loop regions and active sites of proteins), which may participate in conformational changes or substrate binding [52]. Most of the RMSF values of the complexes have two peaks between residues 220–280, which are higher than other peaks (Figure S2). These residues are mainly located in the intracellular loop 3, extracellular loop 3, transmembrane helix 6 and transmembrane helix 7 regions, which are highly flexible and prone to conformational changes. This result is supported by previous research results [53,54]. Furthermore, the dynamic formation and breakage of intermolecular hydrogen bonds observed over the simulation (Figure S3) underscore their pivotal role in mediating specific odorant recognition and binding stability within the odorant-OR complex. This confirms a thermodynamically favorable and highly stable interaction, validating the initial docking results and providing a more robust, dynamic picture of the molecular recognition event. These simulation results collectively confirm the stability of the predicted binding modes and provide a dynamic basis for how lemon volatiles are recognized at the molecular level.

In conclusion, the MD simulation results validated the stability of the predicted odorant-receptor binding modes identified through molecular docking. The consistently stable RMSD profiles confirmed that the receptor-ligand complexes maintained equilibrated conformations throughout the 100 ns simulations. RMSF analysis highlighted the functional flexibility of specific loop and transmembrane regions, which may play adaptive roles in ligand recognition. The dynamic hydrogen bond analysis further revealed that intermolecular hydrogen bonds serve as key stabilizing forces during the binding process. Collectively, these findings provide robust dynamic evidence supporting the proposed molecular mechanism of lemon volatile compound recognition by olfactory receptors.

## 4. Conclusions

This study investigated the composition and variation in volatile compounds in both peels and pulps of six major cultivated lemon varieties using GC-MS and GC-IMS. Differential metabolites were identified through a random forest algorithm to screen characteristic volatile components distinguishing different lemon cultivars. Subsequent molecular docking and molecular dynamics simulations revealed that hydrophobic interactions and weak hydrogen bonding might play crucial roles in the recognition of lemon flavor compounds by olfactory receptors. Notably, amino acid residues including phenylalanine (PHE), tyrosine (TYR), asparagine (ASN), and histidine (HIS) exhibited the most frequent interactions, suggesting their potential significance in mediating odorant-receptor recognition mechanisms. The findings provide substantial insights for varietal discrimination of lemons and elucidate fundamental interaction patterns between aroma molecules and olfactory receptors. This research establishes a theoretical foundation for improving lemon flavor quality optimization and practical applications.

## Figures and Tables

**Table 1:** Sample Information.

ID	Parts	Elaborate	Sample Size
YLF	pulp	Eureka Lemon pulp	3
BEF		Bearss Lemon pulp	3
LSF		Lisbon Lemon pulp	3
FNF		Fino Lemon pulp	3
FLF		Femminello Lemon pulp	3
WEF		Verna Lemon pulp	3
YLP	peel	Eureka Lemon peel	3
BEP		Bearss Lemon peel	3
LSP		Lisbon Lemon peel	3
FNP		Fino Lemon peel	3
FLP		Femminello Lemon peel	3
WEP		Verna Lemon peel	3

**Table 2:** Interactions between differentially expressed metabolites screened by GC-MS analysis and olfactory receptors.

Compound	CAS	Olfactory Receptor	Ref
1-Decanol	112-30-1	OR2J2, OR1G1, OR1G2, OR2W1, OR1A1	[29,30]
Citronellol	106-22-9	OR1D2	[31]
Furan, 2-pentyl-	3777-69-3	——	——
Hexyl propionate	2445-76-3	——	——
Nonanoic acid, methyl ester	1731-84-6	OR1G1, OR1G2	[30]
2-Formyl-5-methylthiophene	13679-70-4	——	——
trans-2-dodecenal	20407-84-5	——	——
cis-3-Hexenyl isovalerate	35154-45-1	——	——
benzyl thiocyanate	3012-37-1	——	——
α-phellandrene	99-83-2	OR2W1	[23]
2-Nonenal	2463-53-8	OR2W1	[23]
(E)-citral	141-27-5	OR1A1, OR4A5, OR52H1, OR52D1, OR51E1, OR51E1P, OR52A3P, OR52A5, OR51B6, OR2W1, OR52N5, OR2T33, OR1D5, OR10A5, OR10A1, OR51I1, OR52W1, OR52W1P, OR6C68, OR10G4, OR2J3, OR51B2, OR51B1P, OR51A2, OR2D2, OR2D1, OR1G1, OR1G2, OR52N4, OR51M1, OR1J1	[32]
Myrcene	123-35-3	OR2W1	[23]
alpha-irone	79-69-6	——	——
D-carvone	2244-16-8	OR1A1, OR5P3, OR10A6, OR5P3, OR8B3, OR2W1	[23,32,33,34]
exyl 2-methylbutanoate	10032-15-2	——	——
(−)-Perillaldehyde	18031-40-8	——	——
2-Ethyl-3-methylpyrazine	15707-23-0	OR5K1	[35]
1,2,4,5-Tetramethylbenzene	95-93-2	——	——
6-Amyl-2-pyrone	27593-23-3	——	——
Maple lactone pyrazine	23747-48-0	OR5K1	[35]
3-hydroxy-4-methyl-5-ethyl-2-furanone	698-10-2	OR8D1, OR8D3	[36]
2-Furanmethanethiol,5-methyl	59303-05-8	——	——
(Z)-2-Decenal	2497-25-8	——	——
Phenol,2-nitro	88-75-5	——	——
2-Acetylthiophene	88-15-3	——	——
β-Ionone	79-77-6	OR1A2, OR5A1, OR5A1P, OR2B3, OR2B3P, OR52D1	[14,16,37]
2-Methoxy-3-methylpyrazine	2847-30-5	——	——

**Table 3:** Differential metabolites screened by GC-IMS analysis and their interactions with olfactory receptors.

Compound	CAS	Olfactory Receptor	References
(E)-2-Pentenal-M	1576-87-0	——	——
methyl acetate-D	79-20-9	——	——
Acetic acid ethyl ester-D	141-78-6	——	——
(Z)-2-Methylpent-2-enal	623-36-9	——	——
2-Propanol-M	67-63-0	——	——
1-Octen-3-one	4312-99-6	OR1A1, OR1A2, OR2W1	[16,23]
2-methyl-2-propenal-M	78-85-3	——	——
1-nonanal-D	124-19-6	OR10S1, OR1D2, OR10G8, OR52A1, OR2W1, OR1A1, OR1A2, OR1G1, OR1G2,	[16,23,29,31,37,38,39]
1-Propanol	71-23-8	——	——
n-Pentanal-M	110-62-3	——	——
Ethyl 2-methy lpropionate	97-62-1	OR2W1	[23]
2,6-Dimethyl-3-ethylpyrazine	13925-07-0	OR5K1	[40]
1-Penten-3-one-D	1629-58-9	——	——
alpha-terpinolene	586-62-9	——	——
(+)-limonene-M	138-86-3	OR2W1, OR1A1, OR1G1, OR1G2	[23,37,41]
Butanal	123-72-8	——	——
1-Pentanol	71-41-0	——	——
α-Terpinene-M	99-86-5	——	——
1-hexanal-D	66-25-1	OR1A1, OR10A2, OR10A2P, OR2J3, OR52N4, OR10C1, OR10C2, OR56B1, OR56B1P, OR10H3, OR5K1, OR4K5, OR5M3, OR8B3, OR5P3, OR2W1, OR8B4, OR8B4P, OR1G1, OR1G2, OR8B8, OR2Z1, OR2Z2, OR2B3, OR2B3P	[29,42]
delta 3-carene-M	13466-78-9	——	——
beta-ocimene-M	13877-91-3	——	——
Propanal-M	123-38-6	——	——
(E)-2-Heptenal	18829-55-5	OR2W1	[23]
3-methylbutyl propanoate	105-68-0	——	——

## Data Availability

The original contributions presented in this study are included in the article/Supplementary Materials. Further inquiries can be directed to the corresponding author.

## Supplementary Materials

The following supporting information can be downloaded at: https://www.mdpi.com/article/10.3390/foods15101710/s1, Figure S1: RMSD results of 20 pairs of receptor-ligand MD; Figure S2: RMSF results of 20 pairs of receptor-ligand MD; Figure S3: Number of hydrogen bonds in 20 pairs of receptor-ligand MD; Table S1: UniProt IDs and docking pocket information for 389 olfactory receptors; Table S2: 202 volatile compounds with rOAV values greater than 1 detected in the sample and their classification; Table S3: Classification and Relative Content of Compounds; Table S4: The volatile components and contents detected by GC-IMS in the peel and flesh of different varieties of lemon; Table S5: The molecular docking binding energy and interaction amino acids of odor molecules and olfactory receptors with significant differences in volatile components in lemon pulp of different varieties; Table S6: The molecular docking binding energy and interaction amino acids of odor molecules and olfactory receptors with significant differences in volatile components in lemon peel of different varieties; Table S7: 20 compounds with different aroma types; Table S8: Detailed information on hydrophobic and hydrogen bonding interactions of 20 OR ligand pairs.
