# Supplementary material for: GC-MS and GC-IMS Comprehensive Analysis of Volatile Compounds in the Peel and Pulp of Six Lemon Varieties and Their Interactions with Olfactory Receptors: Molecular Docking and Molecular Dynamics Simulations Studies
Source: Foods. 2026 May 13;15(10):1710. doi: 10.3390/foods15101710 (PMC13206190; doi:10.3390/foods15101710)
Supplement: Supplementary file 1 [file foods-15-01710-s001.zip › foods-4262595-supplementary.pdf]

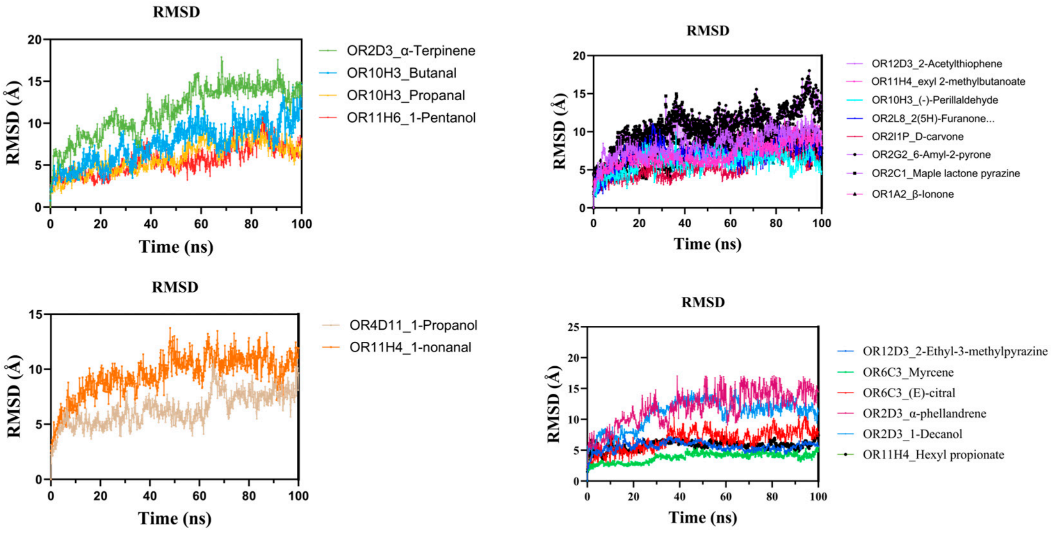


**Figure S1.** RMSD results of 20 pairs of receptor-ligand MD.

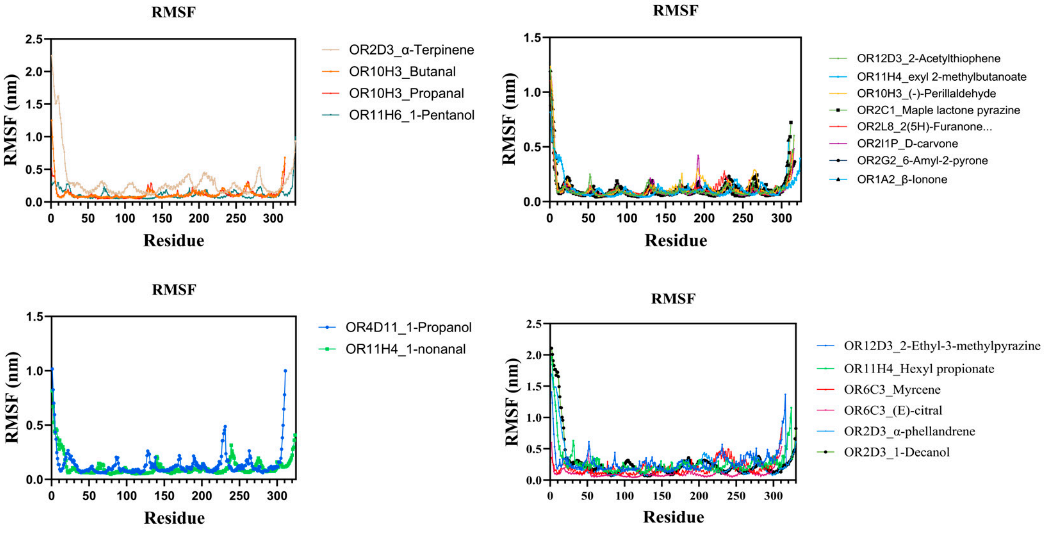


**Figure S2.** RMSF results of 20 pairs of receptor-ligand MD.

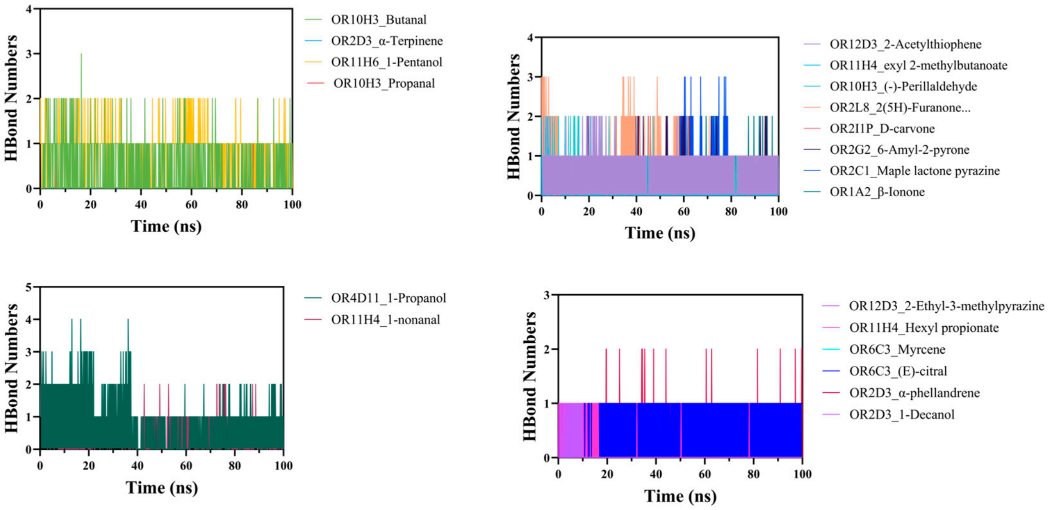


**Figure S3.** Number of hydrogen bonds in 20 pairs of receptor-ligand MD.
